# Natural Capnosanes: Chemical Structures and Bioactivities

**DOI:** 10.3390/ijms27219808

**Published:** 2026-11-01

**Authors:** Szu-Yin Yu, Yi-Hao Lo, Wei-Chiung Chi, Yue-Wen Chuang, Yun-Ting Zeng, Li-Guo Zheng, Su-Ying Chien, Hsi-Wen Sun, Yueh-Wen Liu, Kuang-Nan Tsai, Zhi-Hong Wen, Yu-Chi Tsai, Ping-Jyun Sung

**Affiliations:** 1 Graduate Institute of Natural Products, College of Pharmacy, Kaohsiung Medical University, Kaohsiung 807378, Taiwan; 2 Department of Family Medicine, Zuoying Armed Forces General Hospital, Kaohsiung 813204, Taiwan; a0948060004@mail.ngh.com.tw; 3 Department of Nursing, Meiho University, Pingtung 912009, Taiwan; 4 Department of Food Science, National Quemoy University, Kinmen 892009, Taiwan; 5 Department of Marine Biotechnology & Resources, National Sun Yat-sen University, Kaohsiung 804201, Taiwan; 6 National Museum of Marine Biology & Aquarium, Pingtung 944401, Taiwan; 7 X-Ray Laboratory, Institute of Chemistry, Academia Sinica, Taipei 115201, Taiwan; 8 Department of Chemical Engineering and Biotechnology, Institute of Biochemical and Biomedical Engineering, National Taipei University of Technology, Taipei 106344, Taiwan; 9 Department of Cosmetics and Fashion Styling, Cheng Shiu University, Kaohsiung 833301, Taiwan; 10 Department of Beauty Science, Meiho University, Pingtung 912009, Taiwan; 11 School of Chinese Medicine, College of Chinese Medicine, China Medical University, Taichung 404328, Taiwan; 12 Chinese Medicine Research and Development Center, China Medical University Hospital, Taichung 404394, Taiwan; 13 Ph.D. Program in Pharmaceutical Biotechnology, Fu Jen Catholic University, New Taipei City 242062, Taiwan; 14 Department of Biochemistry and Molecular Medicine, National Dong Hwa University, Hualien 974301, Taiwan; 15 School of Medicine, Kaohsiung Medical University, Kaohsiung 807378, Taiwan; 16 Institute of Marine Ecology and Conservation, National Sun Yat-sen University, Kaohsiung 804201, Taiwan

**Keywords:** capnosane, tobacco, octocoral, diterpenoid, *Nicotiana*, Malacalcyonacea

## Abstract

This review provides a comprehensive overview of the structural characteristics, nomenclature, and biological activities of naturally occurring capnosane diterpenoids (3,7-cyclized cembranoids) reported from 1986 to 2025. The compounds covered in this survey have been isolated primarily from octocorals of the genera *Alcyonium*, *Cespitularia*, *Klyxum*, *Lobophytum*, *Sarcophyton*, and *Sclerophytum*, as well as from the tobacco species *Nicotiana tabacum*. A number of these naturally derived metabolites have demonstrated noteworthy biomedical potential in various biological evaluations.

## 1. Introduction

**Figure 1:**
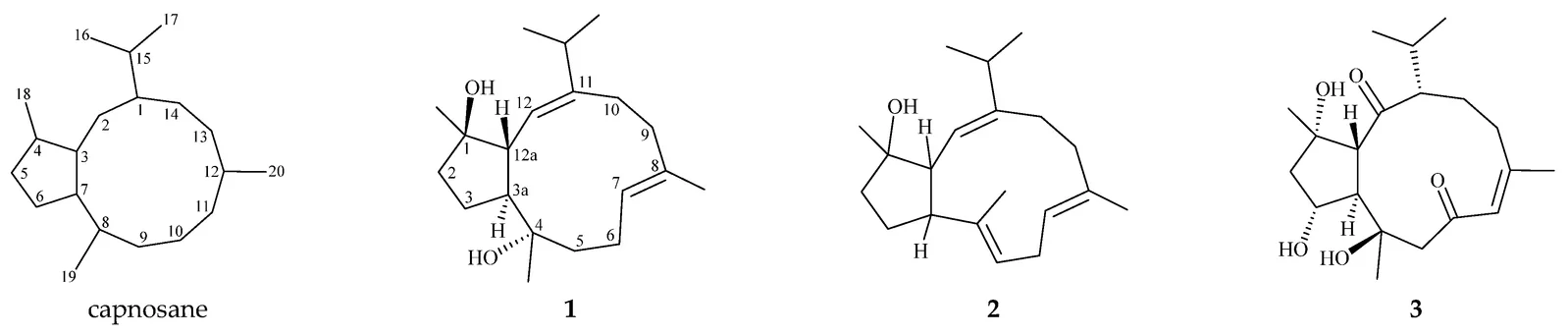
The carbon skeleton of capnosane-type compounds and the structures of (7*E*,11*E*,1R*,3aS*,4S*,12aR*)-11-isopropyl-1,4,8-trimethyl-1,2,3,3a,4,5,6,9,10,12a-decahydrocyclopentacycloundecene-1,4-diol (**1**), (4*E*,7*E*,11*E*)-11-isopropyl-1,4,8-trimethyl-1,2,3, 3a,6,9,10,12a-octahydrocyclopentacycloundecen-1-ol (**2**) (the structure of **2** is tentative), and (1S*,3R*,4S*,6R*,7R*,8R*,11*Z*)-4,6,8-trihydroxy-11-capnosene-2,10-dione (**3**). The carbon numbering system of compounds **1** and **2** differs from that conventionally used for capnosane-type diterpenoids.

Since the first natural capnosane-type diterpenoids (3,7-cyclized cembranoids) (Figure 1) were reported in 1986, specifically, (7*E*,11*E*,1R*,3aS*,4S*,12aR*)-11-isopropyl-1,4,8-trimethyl-1,2,3,3a,4,5,6,9,10,12a-decahydrocyclopentacycloundecene-1,4-diol (**1**) and (4*E*,7*E*,11*E*)-11-isopropyl-1,4,8-trimethyl-1,2,3,3a,6,9,10,12a-octahydrocyclopentacycloundecen-1-ol (**2**) (Figure 1), obtained from an Australian soft coral of the genus *Cespitularia* [1], 71 naturally occurring capnosanes with a bicyclo[9.3.0]tetradecane ring system have been reported. Most of these compounds were obtained from soft corals belonging to the order Malacalcyonacea (phylum Cnidaria, subphylum Anthozoa, class Octocorallia) [2]. Interestingly, a capnosane, (1S*,3R*,4S*,6R*,7R*,8R*,11*Z*)-4,6,8-trihydroxy-11-capnosene-2,10-dione (**3**) (Figure 1), was isolated from the flowers of Greek tobacco, *Nicotiana tabacum*, giving rise to the etymology of “capnosane” from the Greek word καπνός [3].

Biosynthetically, capnosane diterpenoids are derived from the primary 20-carbon isoprenoid precursor geranylgeranyl pyrophosphate (GGPP). Cyclization of GGPP, catalyzed by terpene cyclases, first forms the 14-membered cembrane skeleton via a C1-C14 bond. Subsequent intramolecular cyclization between C-3 and C-7 of the cembrane framework generates the characteristic bicyclic capnosane core. Capnosane-type diterpenoids continue to attract the attention of investigators because of the structural complexity and potential bioactivities (e.g., cytotoxic, anti-fouling, anti-inflammatory, angiogenic, protein tyrosine phosphatase 1B (PTP1B) inhibitory, SARS-CoV-2 M^pro^ inhibitory, and antibacterial activities) associated with numerous compounds of this type. This survey of capnosane-type compounds is presented taxonomically according to genus and species.

## 2. Malacalcyonacea

### 2.1. Alcyonium coralloides (Pallas, 1766) (Family Alcyoniidae)

**Figure 2:**
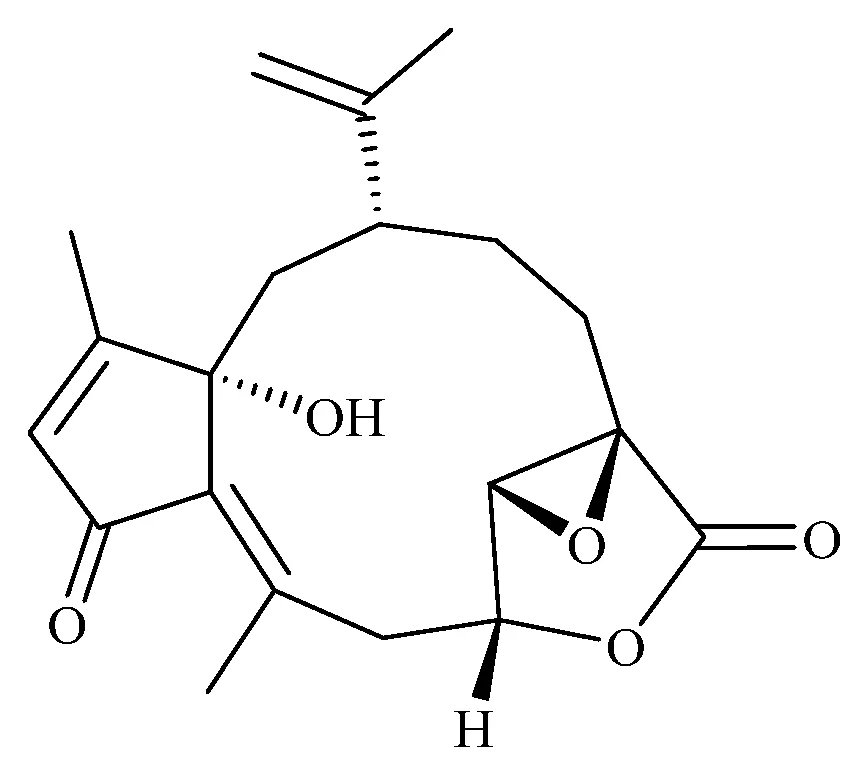
Structure of coralloidolide C (**4**).

Octocorals represent the main natural reservoir of capnosane-type diterpenoids and were subjected to a major taxonomic revision in 2022 [2]. Among these metabolites, coralloidolide C (**4**) (Figure 2) was the first capnosane identified from the soft coral *A. coralloides*, which was collected from the Eastern Pyrenean coastline of the French Mediterranean in 1987 [4]. Its molecular framework and relative stereochemical configuration were established through detailed spectroscopic investigations, including NMR spectra such as HETCOR and INEPT spectra, together with comparisons to previously characterized analogs. Notably, this remains the only documented capnosane-type diterpenoid isolated from a soft coral inhabiting temperate waters rather than tropical or subtropical marine environments.

### 2.2. Cespitularia Species (Milne Edwards & Haime, 1850) (Family Xeniidae)

The first naturally occurring capnosane-type diterpenoids, compounds **1** and **2** (Figure 1), were reported by Bowden and co-workers in 1986 following their isolation from an unidentified *Cespitularia* species collected at East Cay in the Swain Reefs, near Mackay, North Queensland, within Australia’s Great Barrier Reef [1]. Their molecular frameworks were determined using detailed spectroscopic analyses, while the relative configuration of compound **1** was conclusively confirmed via SC-XRD analysis [1].

### 2.3. Klyxum flaccidum (Tixier-Durivault, 1966) (Family Cladiellidae)

**Figure 3:**
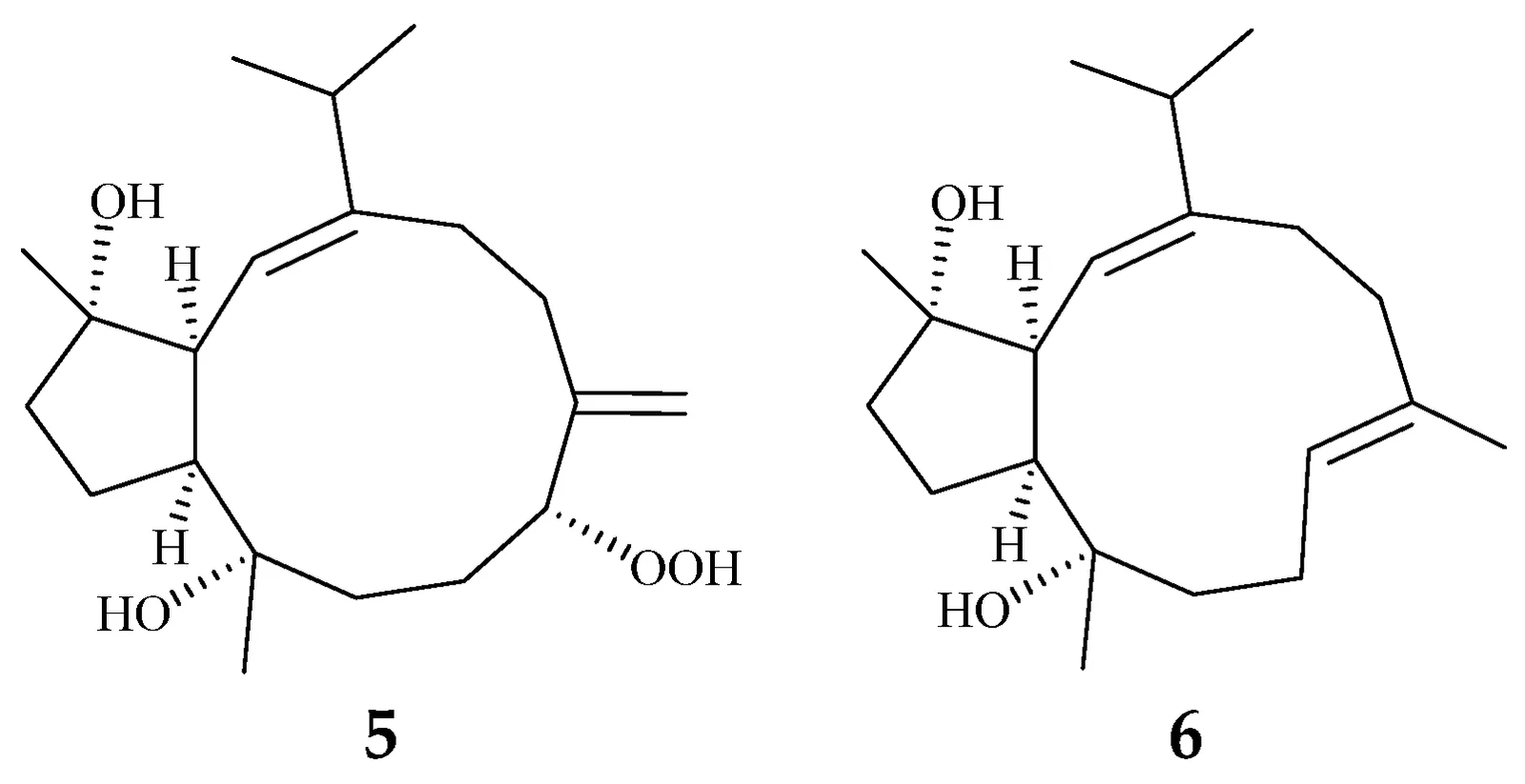
Structures of flaccidenol A (**5**) and 7-*epi*-pavidolide D (**6**).

Flaccidenol A (**5**) and 7-*epi*-pavidolide D (**6**) (Figure 3) were obtained from the soft coral *K*. *flaccidum* collected from around Dongsha (Pratas) Island, the largest island in the South China Sea under Taiwan’s administration [5]. Their chemical structures and relative stereochemical assignments were established through detailed 2D NMR analyses in conjunction with comparisons to previously published spectroscopic data [5,6].

Evaluation of their cytotoxicity revealed that compound **5** was considerably more potent than **6** against human lung adenocarcinoma A549 (IC_50_ = 9.7 vs. 28.6 μg/mL), human colorectal adenocarcinoma DLD-1 (IC_50_ = 6.0 vs. 31.6 μg/mL), and murine leukemia P388D1 (IC_50_ = 7.2 vs. 30.4 μg/mL) cell lines. The enhanced activity of **5** is likely attributable to its hydroperoxy substituent, suggesting that this functional group contributes significantly to the cytotoxic properties of capnosane diterpenoids. To date, compound **5** remains the only reported member of this class to contain a hydroperoxy moiety.

### 2.4. Lobophytum pauciflorum (Ehrenberg, 1834) (Family Sarcophytidae)

**Figure 4:**
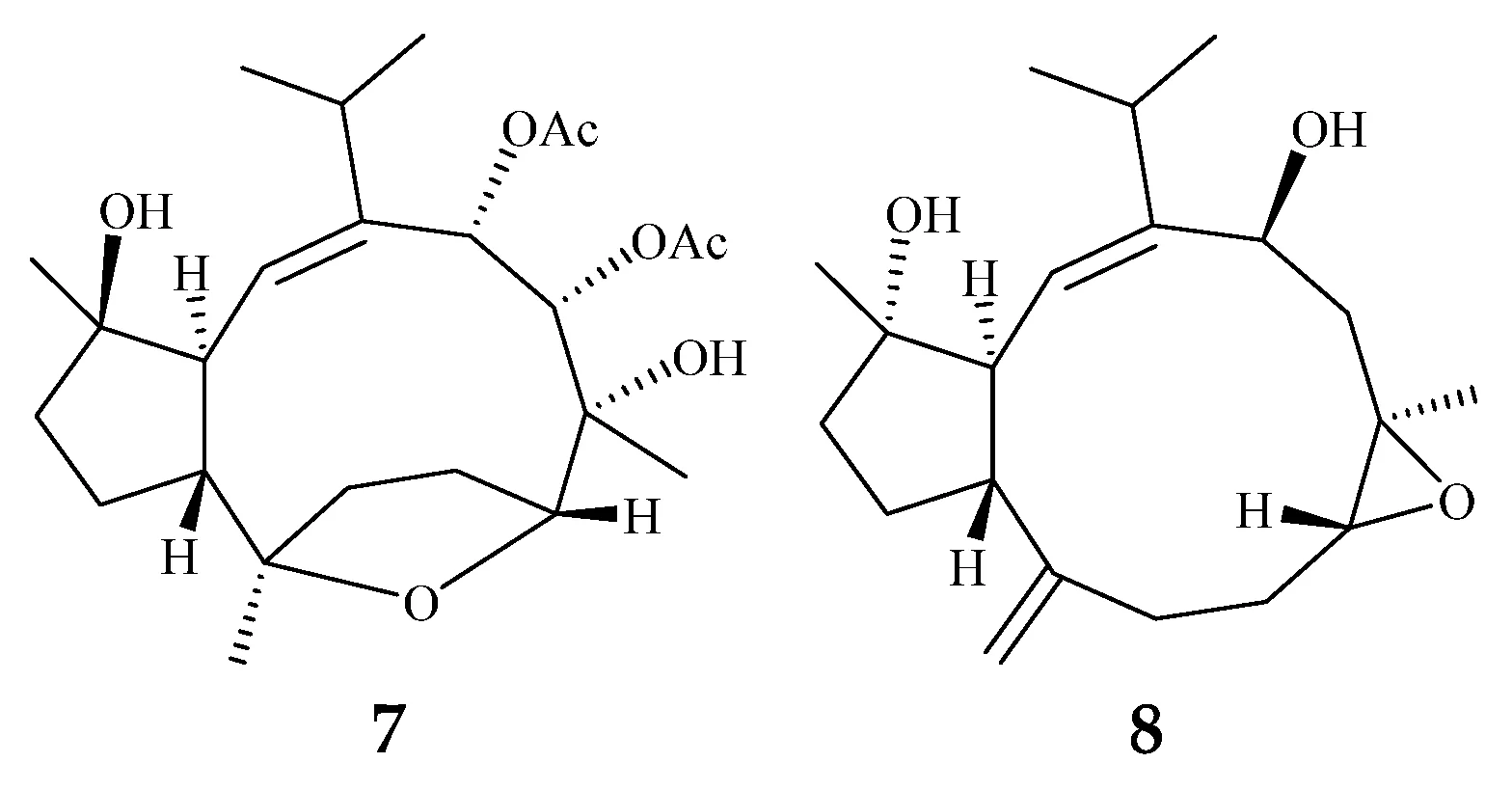
Structures of capnosane **7** and sarcophyolide D (**8**).

A capnosane diterpenoid, **7**, together with sarcophyolide D (**8**) [7] (Figure 4), was isolated from the soft coral *L. pauciflorum* collected from the Yongle Islands of the Xisha Archipelago in the South China Sea [8]. The planar structure of **7** was established using comprehensive spectroscopic analyses, while its absolute configuration was determined through DP4+ quantum chemical calculations in combination with ECD spectroscopy. In a zebrafish inflammation model, compound **7** exhibited moderate anti-inflammatory activity, producing an inhibition rate of 37.4% at a concentration of 20 μM [8].

The authors cited an earlier publication to support the claim that capnosane-type diterpenoids had previously been reported from soft corals of the genus *Lobophytum* [9]. However, examination of the cited literature reveals that the referenced study actually described these metabolites from *Sinularia humilis* rather than from a *Lobophytum* species [9]. Consequently, both the citation and the corresponding statement should be revised to accurately reflect the original source. Furthermore, the structure assigned to the known capnosane sarcophyolide D (**8**) requires correction. Based on the available structural evidence, the hydroxy substituent at C-4 should be depicted with an *α*-orientation, as illustrated in Figure 4 [7,8].

### 2.5. Lobophytum Species (Marenzeller, 1886)

**Figure 5:**
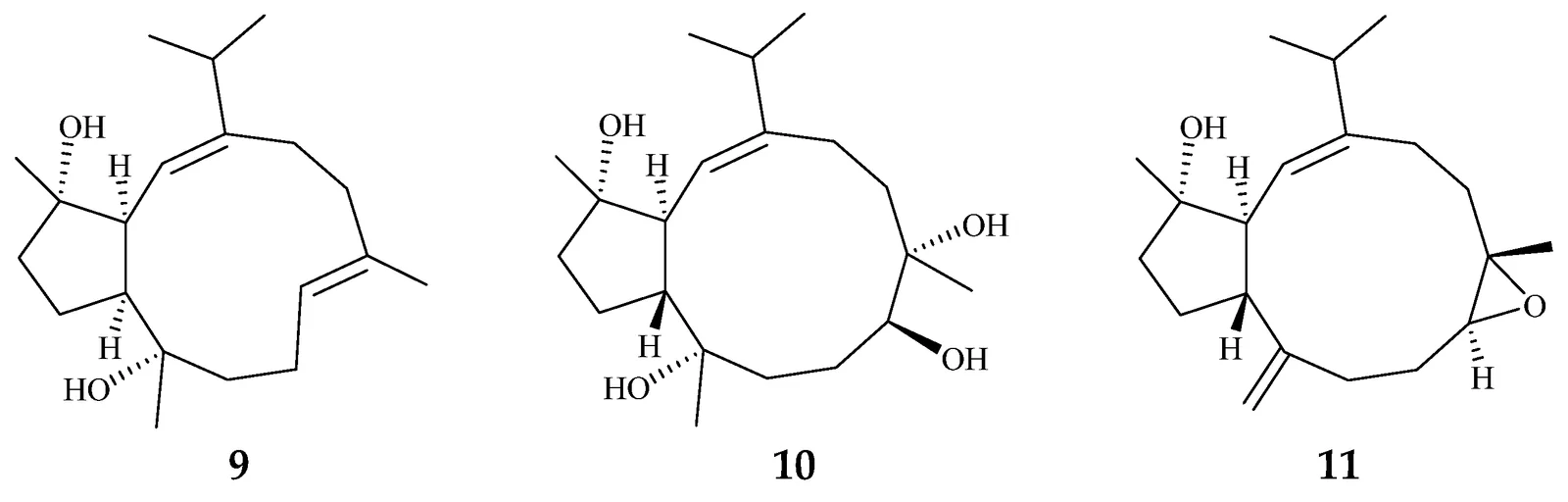
Structures of lobophytrols A–C (**9**–**11**).

Three capnosane diterpenoids, designated lobophytrols A–C (**9**–**11**) (Figure 5), were obtained from a *Lobophytum* sp. soft coral collected off Weizhou Island in Guangxi, China [10]. Their structures were elucidated primarily through spectroscopic analyses. A comparison of the NMR data revealed that lobophytrol A (**9**) has the same structure as 7-*epi*-pavidolide D (**6**) [5]. Although the reported optical rotations differ in sign—compound **9** exhibited ${\left[\alpha \right]}_{D}^{20}$ +1.5 (*c* 0.13, CH_3_OH), whereas compound **6** showed ${\left[\alpha \right]}_{D}^{25}$ –3.8 (*c* 2.77, CHCl_3_)—both values are very close to zero and were measured under different experimental conditions. Consequently, it remains uncertain whether these compounds constitute an enantiomeric pair. Despite this ambiguity, the available spectroscopic evidence supports the finding that they possess the same relative stereochemical configuration.

### 2.6. Sarcophyton boettgeri (Schenk, 1896) (Family Sarcophytidae)

**Figure 6:**
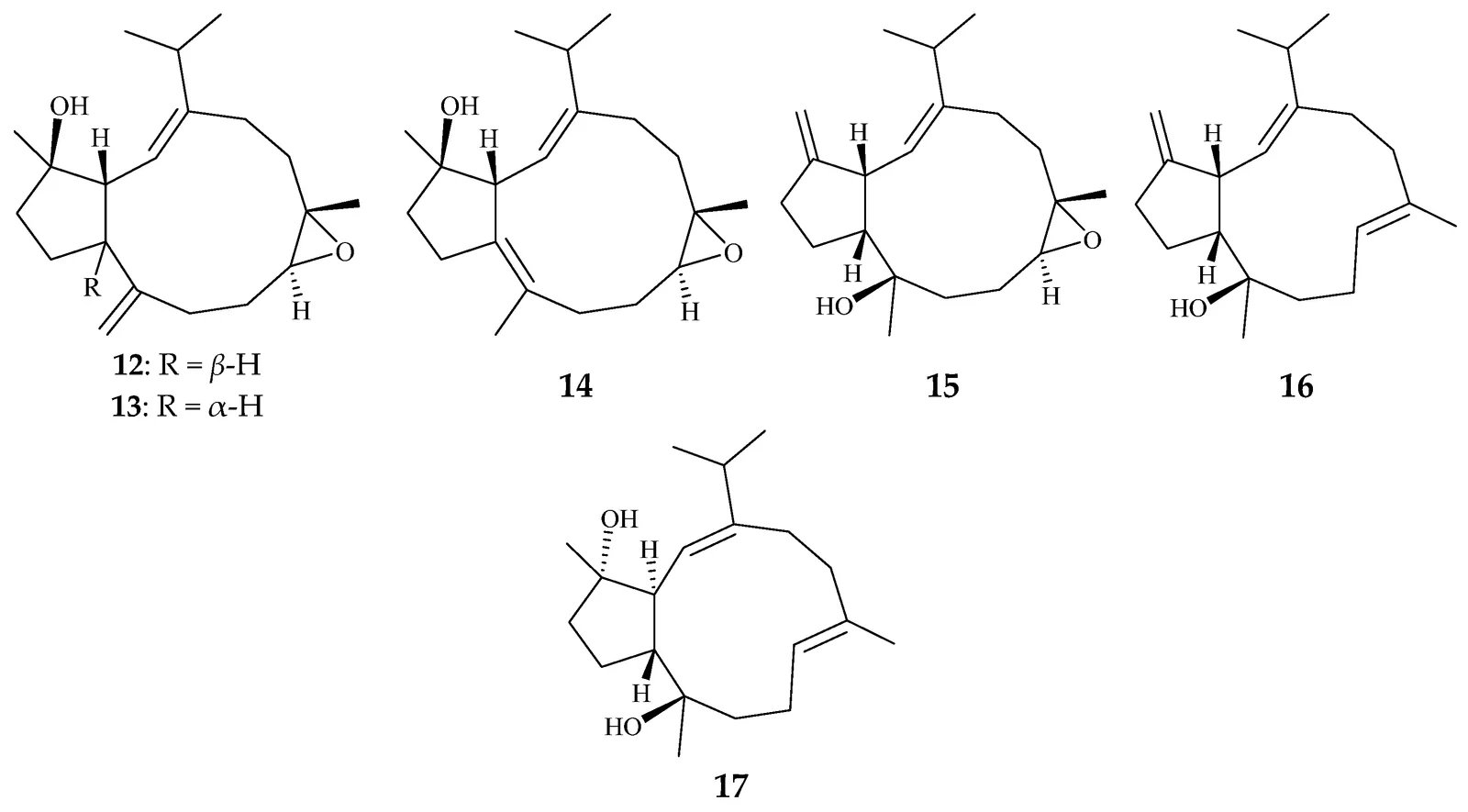
Structures of sarboettgerins A−E (**12**−**16**) and pavidolide D (**17**).

Over the last forty years, octocorals belonging to the genus *Sarcophyton* have been identified as the richest natural reservoirs of capnosane diterpenoids. Chemical studies of *S. boettgeri*, a species broadly distributed across the Indo-Pacific region, resulted in the isolation of five metabolites, sarboettgerins A–E (**12**–**16**), along with pavidolide D (**17**) (Figure 6) [6,11]. The planar structure and absolute stereochemistry of sarboettgerin A (**12**) were established through extensive spectroscopic characterization and definitively confirmed via SC-XRD analysis, which gave a Flack parameter of −0.06. The structures and absolute configurations of sarboettgerins B–E (**13**–**16**) were assigned using spectroscopic analyses and further validated via QM-NMR calculations employing the DP4+ probability method. In biological evaluation, sarboettgerins E (**16**) and D (**15**) exhibited anti-neuroinflammatory potential by suppressing LPS-stimulated NO production in BV-2 murine microglial cells at 40 μM, with NO production reduced to 13.30 and 39.03%, respectively. The greater inhibitory activity of **16** suggests that the *E*-configured olefin between C-11 and C-12 may contribute to enhancing anti-neuroinflammatory activity [11].

### 2.7. Sarcophyton cinereum (Tixier-Durivault, 1946)

**Figure 7:**
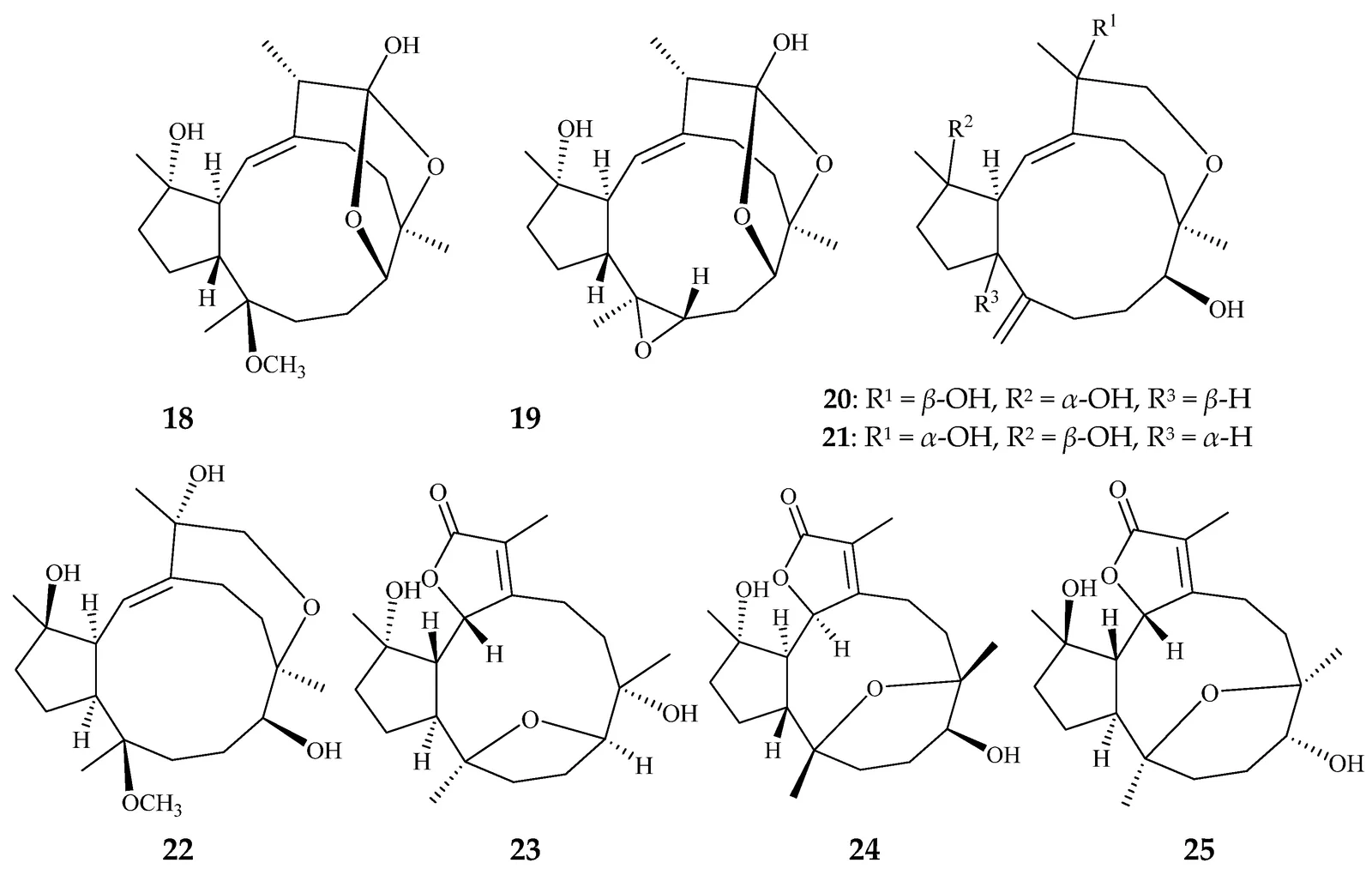
Structures of sarcocinerenoids A−H (**18**−**25**).

Chemical investigation of the soft coral *S. cinereum*, collected from Xisha Island in the South China Sea, led to the isolation of eight highly oxygenated capnosane diterpenoids, designated sarcocinerenoids A–H (**18**–**25**) (Figure 7) [12]. Their molecular structures were established primarily through comprehensive spectroscopic analyses. The absolute stereochemistries of sarcocinerenoids A–C (**18**–**20**), F (**23**), and G (**24**) were unequivocally confirmed via SC-XRD analysis, while those of sarcocinerenoids D (**21**) and H (**25**) were assigned using TDDFT-ECD calculations. The stereochemistry of sarcocinerenoid E (**22**) was resolved by employing the DP4+ method [12]. Among capnosane diterpenoids, sarcocinerenoids A–E (**18**–**22**) are distinguished by the presence of a rare oxepane ring. Interestingly, despite exhibiting similarly small positive optical rotation values (${\left[\alpha \right]}_{D}^{25}$ +4 for **24** and +5 for **25** in MeOH), sarcocinerenoids G (**24**) and H (**25**) were independently demonstrated to possess opposite absolute configurations via SC-XRD analysis and TDDFT-ECD calculations, respectively, highlighting that optical rotation alone may be insufficient for reliable stereochemical determination when the observed values are close to zero. Biological assays further revealed that sarcocinerenoids A (**18**) and D (**21**) promoted angiogenesis in a zebrafish model [12].

### 2.8. Sarcophyton crassocaule (Moser, 1919)

**Figure 8:**
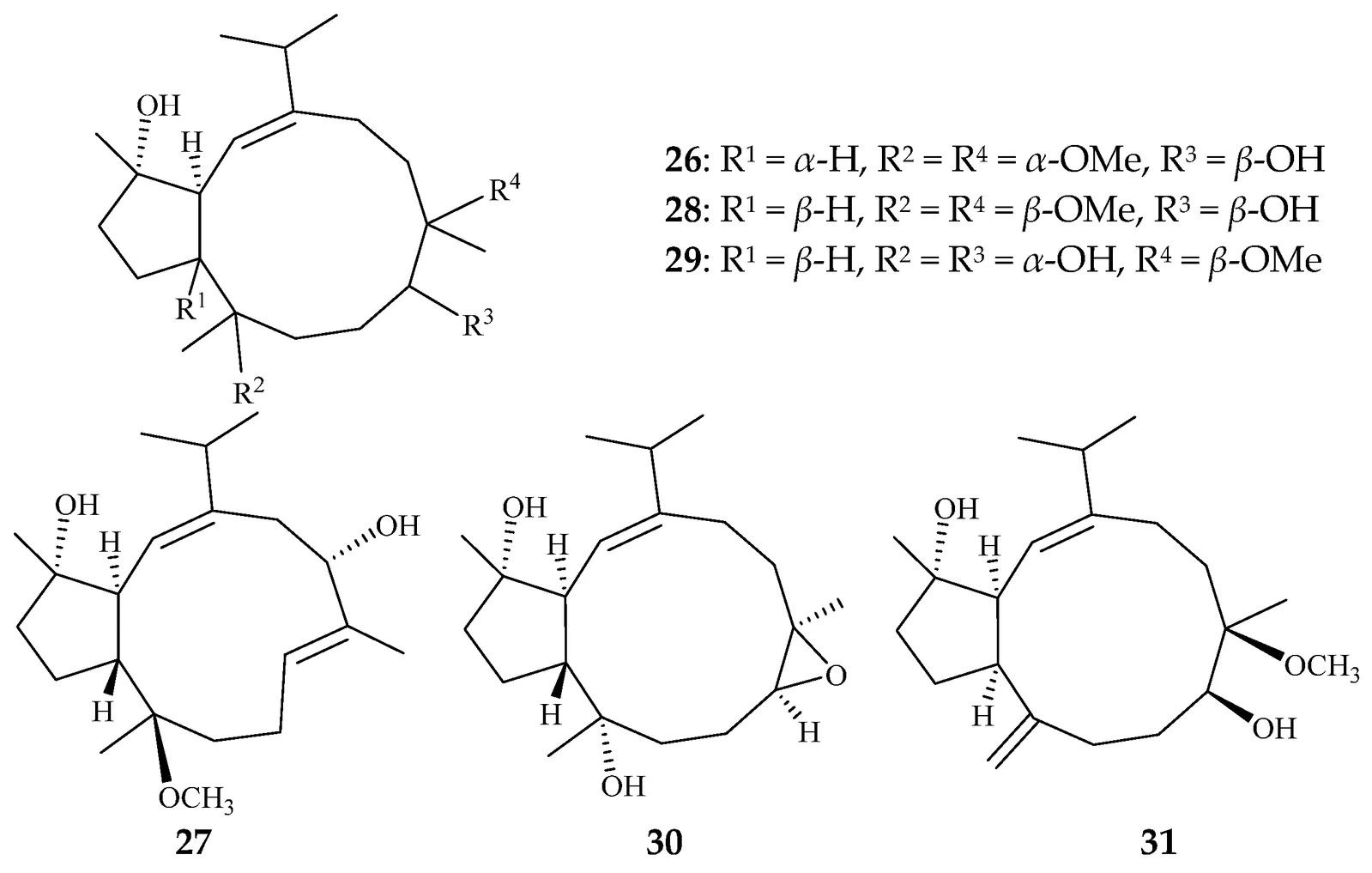
Structures of sarcocrassolins A−F (**26**−**31**).

Six capnosane diterpenoids, sarcocrassolins A–F (**26**–**31**) (Figure 8), together with pavidolide D (**17**) (Figure 6) [6,13], were isolated from the octocoral *S. crassocaule*, collected from the Nansha Islands in the South China Sea [13]. Their structures were established through extensive spectroscopic analyses, supported by QM-NMR calculations. The structure and stereochemistry of sarcocrassolin B (**27**) were further verified via SC-XRD analysis, which afforded a Flack parameter of –0.3. Biological evaluation demonstrated that sarcocrassolins D (**29**) and E (**30**) suppressed LPS-induced inflammatory responses in RAW264.7 macrophages, with IC_50_ values of 76.8 and 93.0 μM, respectively.

**Figure 9:**
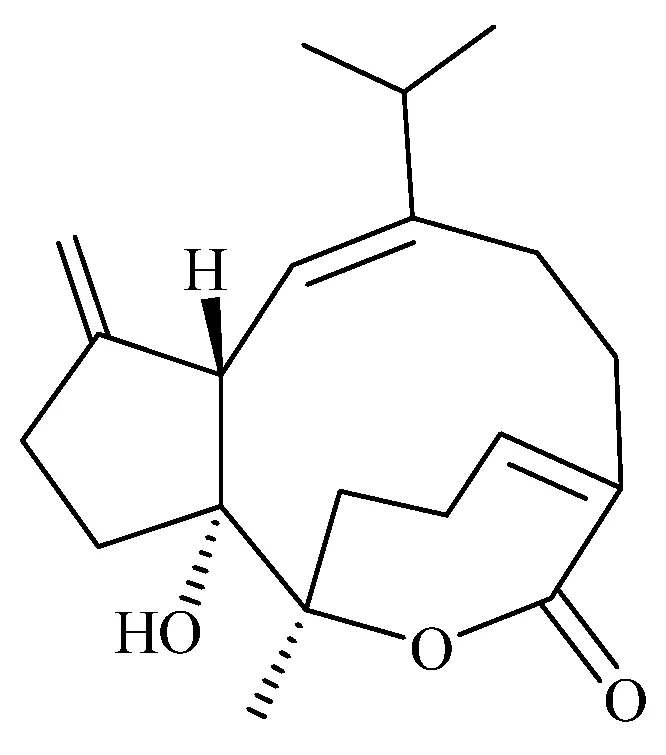
Structure of sarsolilide A (**32**).

Another representative capnosane diterpenoid, sarsolilide A (**32**) (Figure 9), was originally isolated from *Sarcophyton solidum* collected near Xisha Island in the South China Sea [14] and was later identified in *S. crassocaule* from the same region [15]. Its planar structure and relative stereochemistry were initially established through detailed spectroscopic analyses and conclusively confirmed through SC-XRD analysis [14]. A subsequent study reported the occurrence of sarsolilide A (**32**) in *Sarcophyton glaucum* collected from Taiwan, allowing its absolute configuration to be unambiguously assigned through SC-XRD analysis, with a Flack parameter of 0.08 [16]. In pharmacological assays, sarsolilide A (**32**) displayed significant inhibitory activity against protein tyrosine phosphatase 1B (PTP1B), with an IC_50_ value of 6.8 μM [17].

### 2.9. Sarcophyton elegans (Moser, 1919)

**Figure 10:**
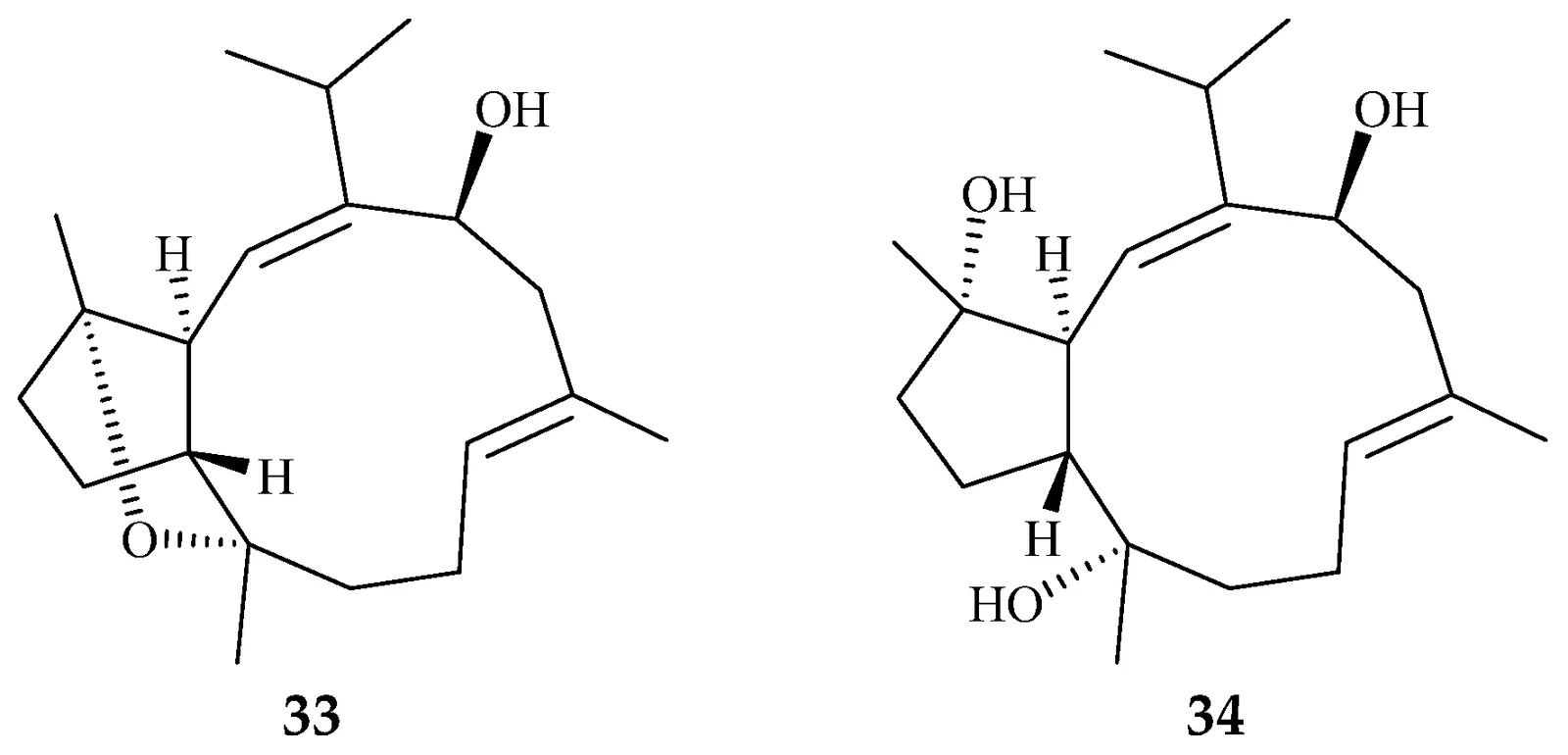
Structures of sarcophyolides B (**33**) and C (**34**).

In 2013, three capnosane diterpenoids, sarcophyolides B (**33**), C (**34**) (Figure 10), and D (**8**) (Figure 4), were isolated from the octocoral *S. elegans* collected from the waters surrounding Xidao Island, China [7]. Their planar structures were elucidated through comprehensive spectroscopic analyses. The absolute configuration of sarcophyolide B (**33**) was established using Mosher’s method, whereas that of sarcophyolide C (**34**) was determined via chemical conversion from **33** under acidic conditions [7]. The structures and absolute configurations of compounds **33** and **34** were unambiguously established through SC-XRD analysis, while the absolute configuration of compound **8** was determined from CD spectroscopic data in conjunction with the *p*-helicity rule [7]. Sarcophyolide B (**33**) exhibited cytotoxicity against A2780 human ovarian cancer cells (IC_50_ = 2.92 μM), whereas sarcophyolide C (**34**) was inactive (IC_50_ > 10 μg/mL). The difference in cytotoxicity suggests that the ether bridge between C-4 and C-8 may play an important role in enhancing cytotoxic activity [7].

**Figure 11:**
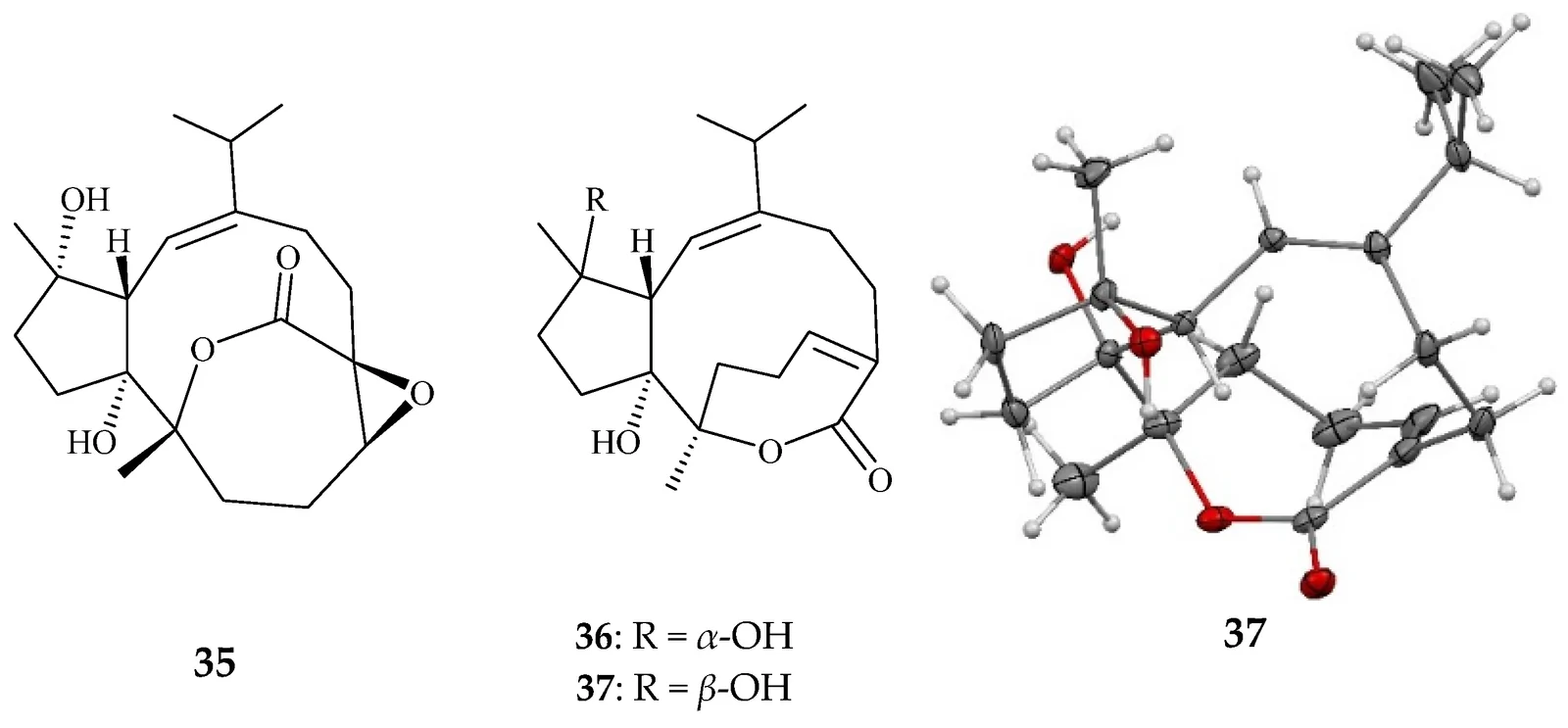
Structures of sarcophelegan A (**35**) and sarsolilides B (**36**) and C (**37**) and a computer-generated ORTEP diagram of sarsolilide C (**37**).

Yin’s group reported the isolation of three 3-hydroxycapnosanes possessing an *ε*-lactone moiety from the octocoral *S. elegans* collected from the Xisha Islands in the South China Sea [18]. The isolates comprised sarcophelegan A (**35**) [18], together with sarsolilides B (**36**) and C (**37**) (Figure 11), which had originally been identified from *S. trocheliophorum* collected near the Nansha Islands, South China Sea [17]. Notably, the structure and absolute configuration of **35** were established through comprehensive spectroscopic analyses and unequivocally confirmed through SC-XRD analysis, with a Flack parameter of –0.02 [18].

In contrast, the absolute configuration of capnosane **36** (sarsolilide B) was initially assigned on the basis of spectroscopic analyses in combination with TDDFT-ECD calculations [17], with the stereochemical assignment of **37** being further supported by a comparison of its spectrum with that of **36** [17]. While such approaches are widely accepted, their conclusions inherently rely on computational models and spectral similarity. Importantly, subsequent isolation of sarsolilide C (**37**) from *S*. *glaucum* collected in Taiwan enabled an unambiguous determination of its absolute configuration through SC-XRD analysis, with a Flack parameter of 0.1 (Figure 11) [19], thereby providing rare crystallographic validation of the earlier assignment. Biologically, sarsolilide B (**36**) exhibited moderate in vitro inhibitory activity against PTP1B (IC_50_ = 27.1 μM), whereas sarsolilide C (**37**) showed no appreciable activity at concentrations up to 50 μM (IC_50_ ≥ 50 μM). A comparison of the PTP1B inhibitory activities of sarsolilides A–C (**32**, **36**, and **37**) suggests that the exomethylene group at C-4/C-18 may be an important structural feature contributing to the observed activity, as removal of this functionality in **36** and **37** was accompanied by a substantial reduction in activity relative to that of **32**. Furthermore, the activity appears to be influenced by the stereochemistry at C-4, with the 4*S*-configured compound **36** exhibiting greater inhibitory activity than the corresponding 4*R*-configured compound **37** [17].

### 2.10. Sarcophyton glaucum (Quoy & Gaimard, 1833)

**Figure 12:**
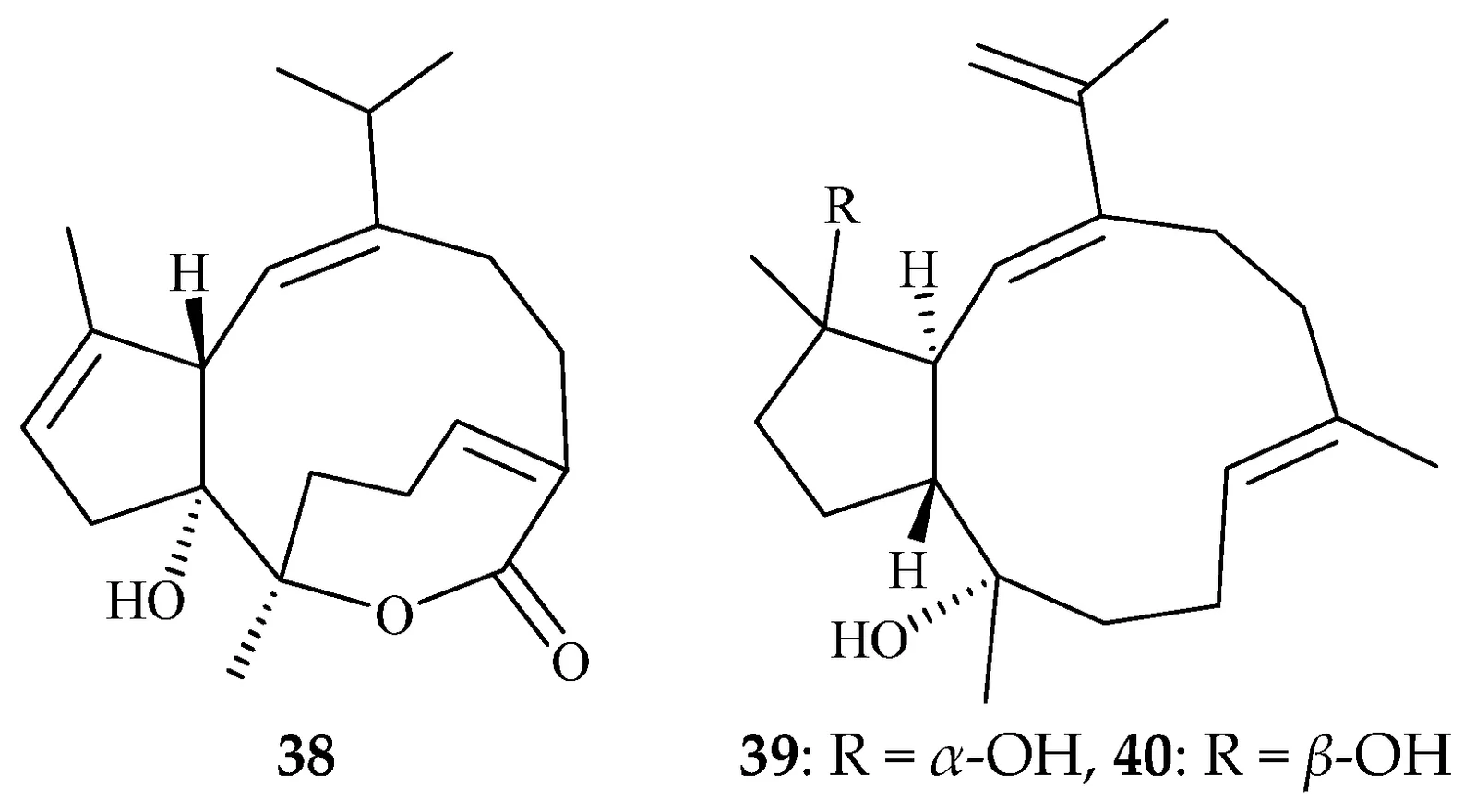
Structures of glacunoid A (**38**) and mililatensols B (**39**) and C (**40**).

Three capnosanes, namely, glacunoid A (**38**) (Figure 12) and sarsolilides A (**32**) (Figure 9) [14,15,16,17] and B (**36**) (Figure 11) [17,18], were isolated from the soft coral *S. glaucum*, collected off the coast of Pingtung County, Taiwan [16]. The structures of **32**, **36**, and **38** were elucidated using spectroscopic methods and by comparing the spectroscopic data with those reported previously. The absolute configuration of compound **38** was further established through an SC-XRD analysis, with a Flack parameter of 0.16 [16].

### 2.11. Sarcophyton mililatensis (Verseveldt & Tursch, 1979)

In the course of ongoing studies aiming to discover structurally novel and bioactive metabolites from the South China Sea octocorals, *S. mililatensis* was collected from Xigu Island, Hainan Province, China. A chemical investigation of this organism resulted in the isolation of two capnosanes, mililatensols B (**39**) and C (**40**) (Figure 12) [20]. Their structures were elucidated through detailed spectroscopic analyses and comparison with literature data, and the absolute configuration of mililatensol B (**39**) was determined through TDDFT-ECD calculations. Furthermore, preliminary molecular docking studies indicated that compounds **39** and **40** possess potential binding affinity to the SARS-CoV-2 M^pro^ [20].

### 2.12. Sarcophyton solidum (Tixier-Durivault, 1958)

In 1992, sarsolilide A (**32**) (Figure 9), the first capnosane-type diterpenoid reported from soft corals of the genus *Sarcophyton*, was isolated from *S. solidum* collected in the South China Sea [14]. Its structure was elucidated through spectroscopic analyses; its planar structure, together with its relative stereochemistry, was confirmed through SC-XRD analysis; and its absolute configuration was further determined through SC-XRD analysis using Cu K*α* radiation [14,16].

Moreover, in 2015, sarsolilides A (**32**) and B (**36**) (Figure 9 and Figure 11) [14,15,16,17,18] were isolated from *S. solidum* collected from the South China Sea (Dongshan Island, Guangdong Province, China) [21]. Although the authors reported that the absolute configuration of sarsolilide B (**36**) was determined for the first time using SC-XRD analysis and Cu K*α* radiation, this assignment should be treated with caution. The Flack value reported for the X-ray structure of compound **36** was −2.2 [21], which does not support a reliable determination of the absolute configuration. Therefore, a re-evaluation of the SC-XRD data and their interpretation for this compound is warranted. Capnosanes **32** and **36** were claimed to be chemotaxonomic markers for the species *S. solidum* [21].

### 2.13. Sarcophyton subviride (Tixier-Durivault, 1958)

**Figure 13:**
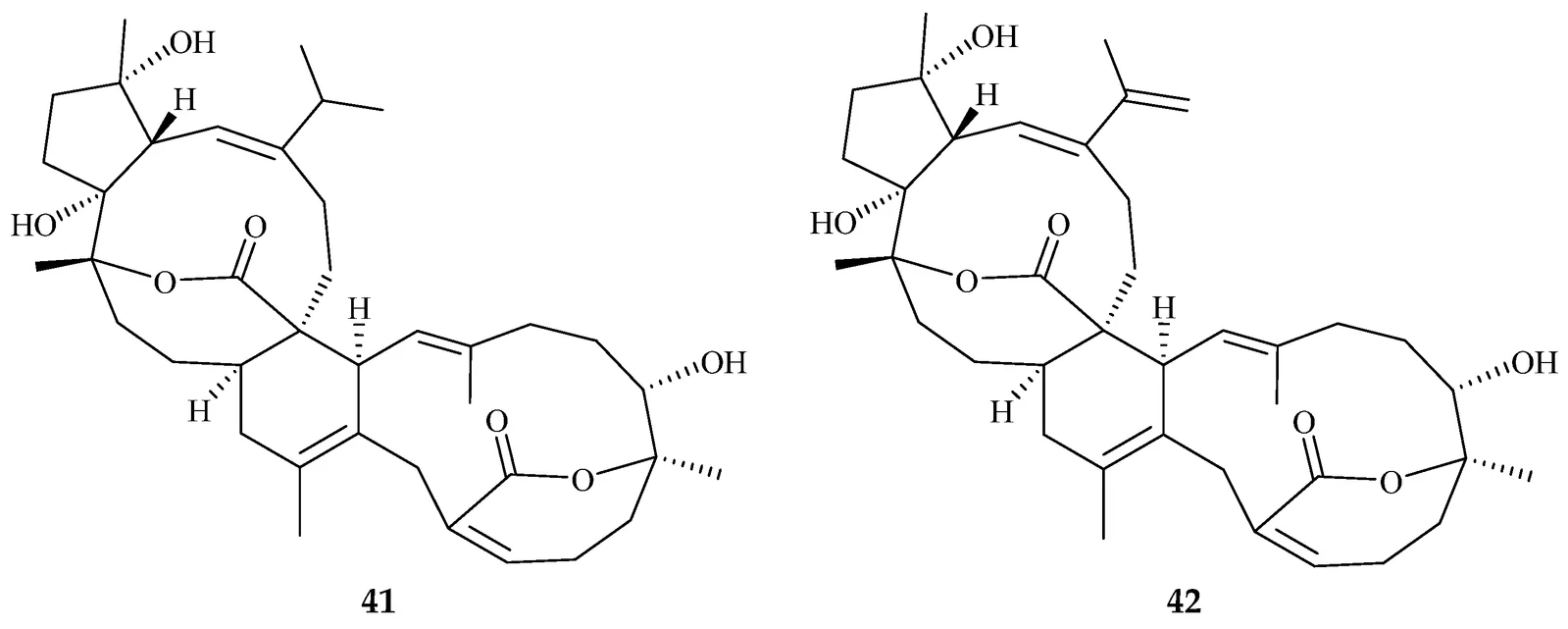
Structures of bissubvilides A (**41**) and B (**42**).

Two cembrane−capnosane heterodimers, bissubvilides A (**41**) and B (**42**) (Figure 13), together with sarsolilide B (**36**) [16,17,18,21] (Figure 11), were isolated from the soft coral *S. subviride* collected from the coast of Xisha Island in the South China Sea [22]. The structures and absolute configurations of compounds **41** and **42** were established through comprehensive spectroscopic analyses in combination with TDDFT-ECD and DFT-NMR calculations. Notably, heterodimers **41** and **42** represent the first examples of biscembranoid-like skeletons presumed to arise from a Diels−Alder cycloaddition between a cembrane-type diene and a capnosane-type dienophile [22].

### 2.14. Sarcophyton trocheliphorum (von Marenzeller, 1886)

**Figure 14:**
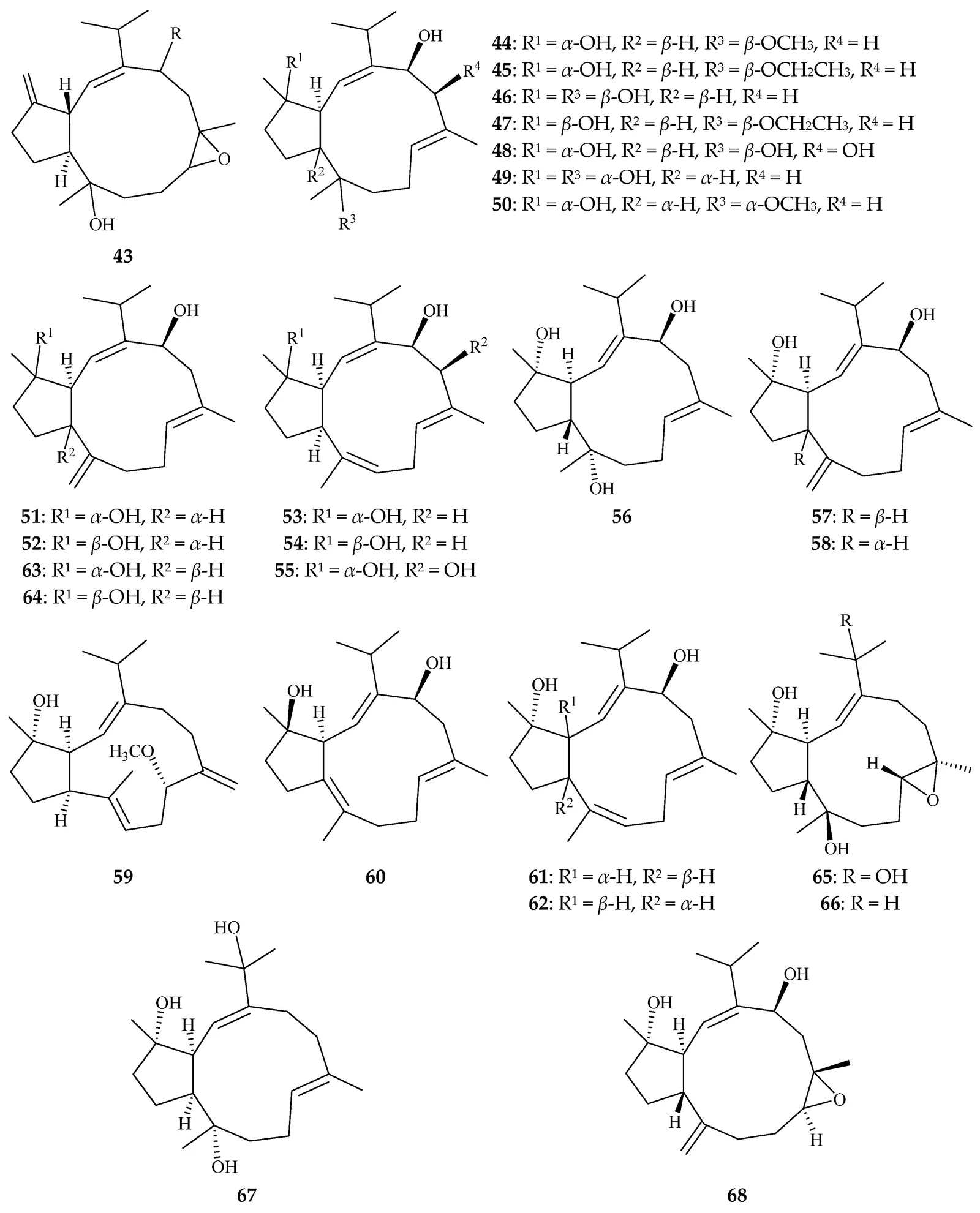
Structures of capnosane **43** (R = OH) and its acetylated derivative (R = OAc), trocheliophols A–S (**44**–**62**), sarcophytol L (**63**), 4-*epi*-sarcophytol L (**64**), sarcophytrols A–C (**65**–**67**), and sartrocheliol A (**68**).

Venkateswarlu’s group isolated an unnamed capnosane, **43**, from the octocoral *S. trocheliphorum*, collected from the Andaman and Nicobar Islands [23]. Due to the difficulty in obtaining compound **43** in a pure form, its structure was elucidated indirectly. A mixture containing **43** and fatty alcohols was subjected to acetylation, and spectroscopic analysis of the resulting acetylated derivative of **43** (Figure 14) allowed the planar structure of capnosane **43** to be established. However, the stereochemistry of the stereogenic centers at C-8, C-11, C-12, and C-14 in **43** was unresolved [23].

As the genus *Sarcophyton* represents the most prolific source and a major geographic reservoir of capnosane-type diterpenoids, two capnosanes, sarsolilides B (**36**) and C (**37**) (Figure 11), were isolated from *S. trocheliophorum* collected at Yalong Bay, Hainan Province, China [17]. The structures of compounds **36** and **37** were elucidated through spectroscopic analysis, and the absolute configuration of sarsolilide B (**36**) was established through TDDFT-ECD calculations.

A total of 23 capnosanes, namely, trocheliophols A–S (**44**–**62**) [24], together with sarcophytol L (**63**) [25,26], 4-*epi*-sarcophytol L (**64**) (Figure 14) [26], and sarcophyolides B (**33**) and C (**34**) (Figure 10) [7], were isolated from the octocoral *S*. *trocheliophorum* collected from the inner coral reef of Weizhou Island, Southwestern China (Figure 14) [24]. The known metabolites were identified as sarcophytol L (**63**) [25,26], 4-*epi*-sarcophytol L (**64**) (Figure 14) [26], and sarcophyolides B (**33**) and C (**34**) (Figure 10) [7]. The structures of compounds **44**–**62** were elucidated through comprehensive spectroscopic analyses. The stereochemical assignments of capnosanes **44** (trocheliophol A), **45** (trocheliophol B), and **62** (trocheliophol S) were unambiguously established through SC-XRD analysis, with Flack parameters of x = –0.03, –0.13, and 0.1, respectively [24]. The configurations of capnosanes **48** (trocheliophol E) and **55** (trocheliophol L) were determined using Snatzke’s method, whereas those of capnosanes **49** (trocheliophol F), **56** (trocheliophol M), and **58** (trocheliophol O) were assigned using Mosher’s method [24]. The authors proposed that compounds bearing ethoxy substituents, such as capnosanes **45** and **47** (trocheliophol D), may represent extraction artifacts, whereas the natural origin of methoxy-containing analogs, including **44**, **50** (trocheliophol G), and **59** (trocheliophol P), remains unclear [24]. Notably, among these five compounds, the ethoxy or methoxy substituents in all compounds, except for **59**, are located specifically at C-8, suggesting a remarkable degree of structural consistency. Moreover, ethanol was not used as a solvent during the extraction or isolation procedures. These observations therefore argue against the possibility that compounds **44**, **45**, **47**, and **50** are extraction artifacts and support their consideration as naturally occurring metabolites.

Capnosanes **51** (trocheliophol H), **52** (trocheliophol I), **55**, **57** (trocheliophol N), **58,** and **61**–**64** (trocheliophols R and S, sarcophytol L, and 4-*epi*-sarcophytol L) were reported to exhibit antibacterial activity against the Gram-negative bacteria *Xanthomonas vesicatoria*, *Agrobacterium tumefaciens*, and *Pseudomonas lachrymans*, as well as the Gram-positive bacteria *Bacillus subtilis* and *Staphylococcus aureus*, with MIC values in the range of 8–32 μg/mL. A comparison of trocheliophols R (**61**) and S (**62**) suggested that differences in the relative orientations of H-3 and H-7 may contribute to the observed variation in antibacterial activity [24]. Furthermore, trocheliophols H (**51**) and I (**52**) showed greater antibacterial activity against the tested strains than their analogs, trocheliophols F (**49**) and G (**50**), respectively. Taken together, these findings suggest that the C-8/C-19 exo-methylene group may be an important structural feature contributing to the antibacterial activity of capnosane-type diterpenoids [27].

Among these analogs, trocheliophol S (**62**), which possesses an H-3*β* orientation, showed stronger inhibitory activity against the tested bacterial strains [24]. It should be noted that sarcophytol L (**63**) is an artifact first reported by Kobayashi and Osabe [25], rather than by Kobayashi and Nakano [26], as stated by the authors. Subsequent studies demonstrated that **63** can be generated from its precursor, sarcophytol A, via an intramolecular cyclization reaction in CHCl_3_ at room temperature [25,26]. Furthermore, the structure of the known artificial compound 4-*epi*-sarcophytol L (**64**) was originally reported in the work of Kobayashi and Nakano [26], where it was reported without being explicitly named. This compound was not first reported in the work of Czarkie’s group [27] as stated by the authors. Notably, the latter publication does not describe the formation of any capnosane-type compounds, whether of natural or synthetic origin. Therefore, a careful re-evaluation and correction of the cited literature are warranted.

In 2015, three capnosane-type diterpenoids, sarcophytrols A–C (**65**–**67**) (Figure 14), were isolated from the soft coral *S. trocheliophorum*, collected from the same marine area as compounds **44**–**62** [28]. The structures of capnosanes **65**–**67** were elucidated through comprehensive spectroscopic analyses, while the absolute configuration of sarcophytrol A (**65**) was established through SC-XRD analysis, with a Flack parameter of *x* = 0.04 [28]. Notably, compounds **65** and **67** feature an isopropyl alcohol moiety, which is seldom observed among capnosane-related metabolites.

Song and colleagues isolated two capnosanes from the octocoral *S. trocheliophorum* collected from Ximao Island, Hainan Province, China: sartrocheliol A (**68**) (Figure 14) [29] and sarcophyolide D (**8**) (Figure 4) [7]. The structure of capnosane **68** was elucidated through a comprehensive spectroscopic analysis, and its absolute configuration was further confirmed through SC-XRD analysis (with a Flack parameter of *x* = 0.05) [29]. In the paper, the structure of sarcophyolide D appears to have been incorrectly represented. In particular, the C-20 methyl group attached to C-12 is presented with an incorrect orientation and should be *α*-oriented, in accordance with the original structural assignment reported in the earlier literature [7].

### 2.15. Sclerophytum humile (van Ofwegen, 2008) (Synonym Sinularia humilis) (Family Sarcophytidae)

**Figure 15:**
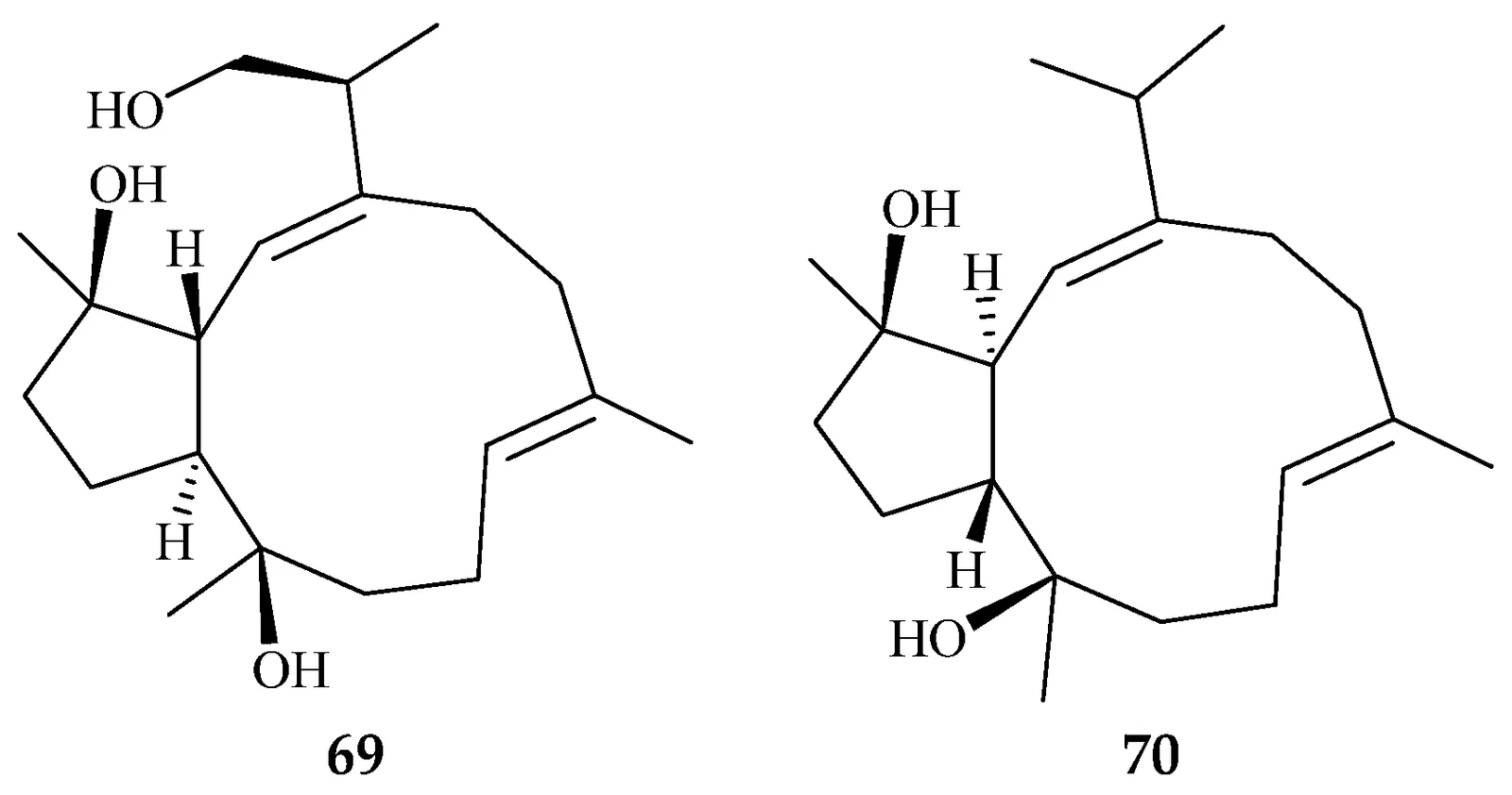
Structures of sinuhumilol A (**69**) and pavidolide E (**70**).

A capnosane, sinuhumilol A (**69**) (Figure 15), was isolated from the soft coral *S. humile*, collected from the South China Sea [9]. The structure of this compound, including its absolute configuration, was elucidated through spectroscopic analysis and DP4+ quantum chemical calculations [9].

### 2.16. Sclerophytum pavidum (Tixier-Durivault, 1970) (Synonym Sinularia pavida)

Two capnosanes, pavidolides D (**17**) and E (**70**) (Figure 6 and Figure 15), were isolated from the soft coral *S. pavidum* collected from Sanya Bay, Hainan Island, China [6]. The structures of compounds **17** and **70** were elucidated through detailed spectroscopic analyses. Notably, pavidolide D (**17**) exhibited a specific rotation of ${\left[\alpha \right]}_{D}$ −90° (*c* = 0.78, CHCl_3_) and was demonstrated to be the enantiomer of compound **1** (${\left[\alpha \right]}_{D}$ +93°, *c* = 0.046, CHCl_3_) (Figure 1) through a comparison of their optical rotation values [1]. In a biological evaluation, pavidolide D (**17**) showed moderate inhibitory activity against the larval settlement of the barnacle *Balanus amphitrite*, with an ED_50_ value of 2.12 μg/mL, while exhibiting low cytotoxicity (LD_50_ > 50 μg/mL). This result suggests that pavidolide D (**17**) may serve as a promising lead for the development of environmentally benign antifouling agents lacking biocidal properties [6]. In this paper, the authors cited an ambiguous reference regarding the structural elucidation of pavidolide D (**17**) [30]. This reference does not describe capnosane-type diterpenoids or related derivatives and therefore does not appear to be relevant to the structural analysis of pavidolide D. This citation is likely a clerical error. The appropriate reference should be [1], which reported capnosane-related diterpenes and is relevant to the structural elucidation discussed in the present work.

### 2.17. Sclerophytum polydactylum (Ehrenberg, 1834) (Synonym Sinularia polydactyla)

**Figure 16:**
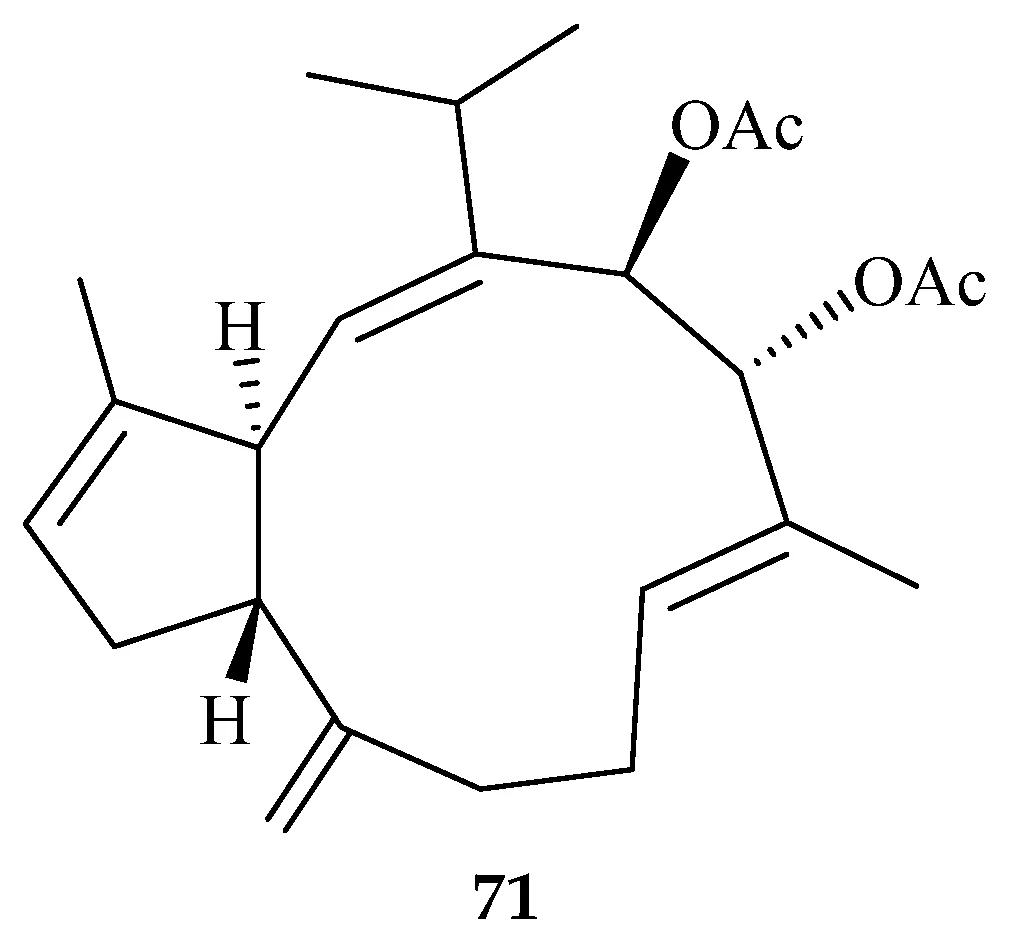
Structure of sinulacetate (**71**).

A capnosane, sinulacetate (**71**) (Figure 16), was isolated from *S. polydactylum* collected from the Xisha Islands, Hainan province, China [31]. The structure of capnosane **71**, including its absolute configuration, was determined using spectroscopic methods and by comparing its NMR data with those reported in the literature and TDDFT-ECD calculations. Sinulacetate (**71**) exhibited promising inhibitory activity against PTP1B, with an IC_50_ value of 51.8 μM.

## 3. Solanales

### Nicotiana tabacum (Linnaeus, 1753) (Family Solanaceae)

Notably, the only capnosane-type diterpenoid of terrestrial origin reported to date, capnosane **3** (Figure 1), was isolated from the flowers of Greek tobacco *N. tabacum* [3]. The structure of **3** was elucidated through spectroscopic analyses, and its relative configuration was further confirmed through SC-XRD analysis of the corresponding benzoate derivative.

## 4. Structural Complexity and Synthetic Challenges

The capnosane scaffold is characterized by an oxidized bicyclo[9.3.0]tetradecane framework that bears multiple stereogenic centers and a variety of oxygenated functionalities. These structural features make capnosanes highly challenging synthetic targets and have continuously attracted attention in the field of synthetic organic chemistry. In addition to the preparation of derivatives for structural elucidation purposes [3,7,23], substantial efforts have been made to develop synthetic strategies for the construction of the densely functionalized and stereochemically complex core skeleton. From a synthetic perspective, innovative methodologies are required to establish the characteristic ring system, together with contiguous stereochemical arrays. Notably, Zhang and Xu reported a concise approach to the stereotetrad core of capnosane diterpenoids, in which the distinctive reactivity of chiral cyclopentane derivatives was utilized to construct the C-3 and C-7 methine stereocenters at the ring junctions [32,33]. In addition, other studies have described either the total synthesis or assembly of key core fragments [34]. Unfortunately, despite these advances, no total synthesis of a naturally occurring capnosane has been achieved to date. To the best of our knowledge, the only reported total synthesis of a capnosane-related compound is that of 6-*epi*-sarsolilide A, an analog of the natural product sarsolilide A, accomplished by Xu and co-workers [33]. However, 6-*epi*-sarsolilide A has not been reported as a naturally occurring compound.

It is also noteworthy that the first synthetic capnosane-type compound was reported as early as 1984 by Shewtsov’s group, who employed isocembrol as a precursor and achieved oxymercuration–demercuration cyclization to afford 1*R*,3*R*,4*R*,7*S*,8*R*-3,7-cyclo-4,8-oxidocembra-11*E*-en, the structure of which was unambiguously confirmed through SC-XRD analysis [35]. Furthermore, through structural studies in natural product chemistry, Kobayashi and Osabe found that the cembrane-type compound sarcophytol A undergoes a ring-closure reaction in CHCl_3_ at room temperature to give the capnosane-type compound sarcophytol L (**63**) (Figure 14) [25,26]. Collectively, these studies have progressively expanded the synthetic accessibility of capnosane diterpenoids and facilitated further investigations into their structural diversification and functionalization.

## 5. Distribution, Biodiversity, and Future Prospects

Between 1986 and 2025, capnosane diterpenoids were reported almost exclusively from marine organisms, with octocorals serving as their main natural source. Among these, species of the genus *Sarcophyton* have yielded the overwhelming majority of known capnosane metabolites. To date, compound **3** remains the sole exception, having been isolated from the terrestrial plant *Nicotiana tabacum*. In contrast, cembrane diterpenoids, which serve as biosynthetic precursors to capnosane-type natural products, are widely distributed in both octocorals and tobacco plants and exhibit a broad range of biological activities [36,37,38]. This distinct distribution pattern suggests that the 3,7-cyclization of cembrane precursors, which leads to the formation of the capnosane skeleton, may be a relatively specific biosynthetic process in octocorals. This distinctive distribution emphasizes the remarkable biosynthetic capacity of tropical and subtropical coral reef ecosystems, which continue to represent an exceptionally rich yet insufficiently explored source of structurally unique marine natural products.

Chemical investigation of capnosanes remains challenging because these metabolites usually possess densely functionalized polycyclic frameworks, a high degree of oxidation, and multiple contiguous stereogenic centers. Consequently, their isolation, structural characterization, and stereochemical assignment require the integration of advanced analytical techniques and continue to drive methodological developments in natural product chemistry and total synthesis.

Future studies are expected to benefit from continued improvements in spectroscopic and computational methodologies, especially the combined application of high-resolution multidimensional NMR techniques and quantum chemical calculations. These advances will facilitate more reliable and efficient structural elucidation while increasing the likelihood of discovering new capnosane analogs with unprecedented skeletal features and valuable biological activities, thereby further broadening their prospects for pharmaceutical research and drug development.

## 6. Conclusions

Over the past four decades, 71 naturally occurring capnosane-type diterpenoids have been identified, with the vast majority originating from marine organisms. Except for compound **3**, which was isolated from the tobacco plant *Nicotiana tabacum*, all known members of this family were obtained from soft corals of the order Malacalcyonacea. This highly restricted taxonomic distribution highlights octocorals as the main natural reservoir of capnosane diterpenoids and underscores the exceptional biosynthetic potential of coral reef ecosystems.

Among the reported 71 metabolites, 24 (33.8%) were found to be active in various biological activity tests (Table 1), and 57 (81.4%) were isolated from species of the genus *Sarcophyton*, demonstrating that this genus is by far the richest source of capnosane diterpenoids discovered to date. The remarkable abundance and structural diversity of these compounds suggest that they possess considerable chemotaxonomic value and may serve as useful chemical markers for *Sarcophyton* species. Nevertheless, despite their taxonomic significance, the ecological roles and physiological functions of capnosane diterpenoids in soft corals remain largely unknown and warrant further investigation. This family of natural products continues to attract considerable attention because of its distinctive polycyclic carbon framework, high degree of oxidation, and diverse biological activities. Continued advances in spectroscopic analysis, crystallographic techniques, and computational chemistry are expected to facilitate the discovery of additional capnosane analogs, improve the accuracy of stereochemical assignment, and provide deeper insight into their biosynthesis, ecological significance, and pharmacological potential. Such efforts will further expand the value of capnosane diterpenoids as promising lead structures for marine natural product research and future drug discovery.

## Figures and Tables

**Table 1:** Bioactive capnosanes from octocorals.

No.	Name	Source and Compound Yield from the Extract	Bioactivity	Ref.
**5**	Flaccidenol A	*Klyxum flaccidum* 0.00125%	IC_50_ (A549, DLD-1, P388D1) = 9.7, 6.0, 7.2 μg/mL	[5]
**6**	7-*epi*-Pavidolide D	*Klyxum flaccidum* 0.00808%	IC_50_ (A549, DLD-1, P388D1) = 28.6, 31.6, 30.4 μg/mL	[5]
**7**		*Lobophytum pauciflorum* 0.00391%	Anti-inflammatory activity, with 37.37% inhibition at 20 μM in a CuSO_4_-induced inflammatory zebrafish model	[8]
**16**	Sarboettgerin E	*Sarcophyton boettgeri* 0.04000%	Exhibited anti-neuroinflammatory activity by reducing LPS-stimulated NO production to 13.3% at 40 μM	[11]
**17**	Pavidolide D	*Sclerophytum pavidum* 0.04000% ^a^	Antifouling activity against the larval settlement of the barnacle *Balanuis amphitrite*, with an ED_50_ value of 2.12 μg/mL	[6,13]
**18**	Sarcocinerenoid A	*Sarcophyton cinereum* 0.00430%	Promoted significant angiogenesis in a zebra fish model at 20 μM	[12]
**21**	Sarcocinerenoid D	*Sarcophyton cinereum* 0.00783%	Promoted angiogenesis in a zebra fish model at 20 μM	[12]
**29**	Sarcocrassolin D	*Sarcophyton crassocaule* 0.03375%	Inhibited NO production in LPS-stimulated RAW264.7 cells, with an IC_50_ value of 76.8 μM	[13]
**30**	Sarcocrassolin E	*Sarcophyton crassocaule* 0.01875%	Inhibited NO production in LPS-stimulated RAW264.7 cells, with an IC_50_ value of 93.0 μM	[13]
**32**	Sarsolide A	*Sarcophyton trocheliophorum* 0.06286%	Exhibited inhibitory activity against PTP1B, with an IC_50_ value of 6.8 μM	[17]
**33**	Sarcophyolide B	*Sarcophyton elegans* 0.00900%	IC_50_ (A2780) = 2.92 μM	[7]
**36**	Sarsolide B	*Sarcophyton trocheliophorum* 0.12857%	Exhibited inhibitory activity against PTP1B, with an IC_50_ value of 27.1 μM	[17]
**39**	Mililatensol B	*Sarcophyton mililatensis* 0.01852%	Potential SARS-CoV-2 M^pro^ inhibitor, with a predicted binding affinity of −7.65 kcal/mol in molecular docking	[20]
**40**	Mililatensol C	*Sarcophyton mililatensis* 0.02296%	Potential SARS-CoV-2 M^pro^ inhibitor, with a predicted binding affinity of −7.03 kcal/mol in molecular docking	[20]
**51** **52**	Trocheliophol H Trocheliophol I	*Sarcophyton trocheliophorum* 0.05143% (for trocheliophol H) and 0.01250% (for trocheliophol I)	These two compounds showed antibacterial activity against *X. vesicatoria*, *A. tumefaciens*, *P. lachrymans*, *B. subtilis*, and *S. aureus;* MIC = 16 μg/mL for all strains	[24]
**55**	Trocheliophol L	*Sarcophyton trocheliophorum* 0.02536%	Antibacterial activity against *X. vesicatoria*, *A. tumefaciens*, *P. lachrymans*, *B. subtilis*, and *S. aureus;* MIC = 32, 32, 32, 32, 16 μg/mL	[24]
**57**	Trocheliophol N	*Sarcophyton trocheliophorum* 0.00679%	Antibacterial activity against *X. vesicatoria*, *A. tumefaciens*, *P. lachrymans*, *B. subtilis*, and *S. aureus;* MIC = 32, 32, 32, 16, 16 μg/mL	[24]
**58**	Trocheliophol O	*Sarcophyton trocheliophorum* 0.00786%	Antibacterial activity against *X. vesicatoria*, *A. tumefaciens*, *P. lachrymans*, *B. subtilis*, and *S. aureus;* MIC = 32, 32, 32, 32, 16 μg/mL	[24]
**61**	Trocheliophol R	*Sarcophyton trocheliophorum* 0.01214%	Antibacterial activity against *X. vesicatoria*, *A. tumefaciens*, *P. lachrymans*, *B. subtilis*, and *S. aureus;* MIC = 16 μg/mL for all strains	[24]
**62**	Trocheliophol S	*Sarcophyton trocheliophorum* 0.01821%	Antibacterial activity against *X. vesicatoria*, *A. tumefaciens*, *P. lachrymans*, *B. subtilis*, and *S. aureus;* MIC = 8 μg/mL for all strains	[24]
**63**	Sarcophytol L	*Sarcophyton trocheliophorum* 0.08214%	Antibacterial activity against *X. vesicatoria*, *A. tumefaciens*, *P. lachrymans*, *B. subtilis*, and *S. aureus;* MIC = 16, 32, 16, 16, 16 μg/mL	[24]
**64**	4-*epi*-Sarcophytol L	*Sarcophyton trocheliophorum* 0.01821%	Antibacterial activity against *X. vesicatoria*, *A. tumefaciens*, *P. lachrymans*, *B. subtilis*, and *S. aureus;* MIC = 32 μg/mL for all strains	[24]
**71**	Sinulacetate	*Sclerophytum polydactylum* 0.00652%	Exhibited inhibitory activity against PTP1B, with an IC_50_ value of 51.8 μM	[31]

^a^ The yield of this compound was derived from the data reported by Peng et al. [13].

## Data Availability

No new data were created or analyzed in this study. Data sharing is not applicable to this article.
